# Turning Trash into Treasure: Silicon Carbide Nanoparticles from Coal Gangue and High-Carbon Waste Materials

**DOI:** 10.3390/molecules30071562

**Published:** 2025-03-31

**Authors:** Kaixing Gao, Yao Zhang, Binghan Wang, Zhuangzhuang Zhang, Sen Luo, Qian Wang, Yanzhong Zhen, Feng Fu, Yucang Liang

**Affiliations:** 1 Shaanxi Key Laboratory of Chemical Reaction Engineering, Collaborative Innovation Center for Clean and Efficient Utilization of Low-Rank Coal of Northern Shaanxi, Research Institute of Comprehensive Energy Industrial Technology, School of Chemistry & Chemical Engineering, Yan’an University, Yan’an 716000, China; gaokxyau@163.com (K.G.); zhangy334@126.com (Y.Z.); 18209283980@163.com (B.W.); luos1715@163.com (S.L.); wqian10032024@163.com (Q.W.); zhenyanzhongyau@163.com (Y.Z.); fufengyau@163.com (F.F.); 2 Institut für Anorganische Chemie, Eberhard Karls Universität Tübingen, Auf der Morgenstelle 18, D-72076 Tübingen, Germany; yucang@163.com

**Keywords:** re-utilization of waste resources, turning trash into treasure, nanostructured silicon carbide, morphological control, carbothermal reduction reaction

## Abstract

To reduce solid waste production and enable the synergistic conversion of solid waste into high-value-added products, we introduce a novel, sustainable, and ecofriendly method. We fabricate nanofiber and nanosheet silicon carbides (SiC) through a carbothermal reduction process. Here, the calcined coal gangue, converted from coal gangue, serves as the silicon source. The carbon sources are the carbonized waste tire residue from waste tires and the pre-treated kerosene co-refining residue. The difference in carbon source results in the alteration of the morphology of the SiC obtained. By optimizing the reaction temperature, time, and mass ratio, the purity of the as-made SiC products with nanofiber-like and nanosheet-like shapes can reach 98%. Based on the influence of synthetic conditions and the results calculated from the change in the Gibbs free energy of the reactions, two mechanisms for SiC formation are proposed, namely the reaction of intermediate SiO with CO to form SiC-nuclei-driven nanofibrous SiC and the SiO-deposited carbon surface to fabricate nuclei-induced polymorphic SiC (dominant nanosheets). This work provides a constructive strategy for preparing nanostructured SiC, thereby achieving “turning trash into treasure” and broadening the sustainable utilization and development of solid wastes.

## 1. Introduction

The consecutive expansion of the global economy is contributing significantly to the ongoing demand for energy worldwide [1]. Public data indicate that China’s raw coal production, as the world’s largest producer and consumer of coal, reached 4.56 billion tons in 2023, maintaining its position as the top producer globally [2]. In 2024, China’s coal production increased to 4.71 billion tons, accounting for approximately 53.8% of the global total. Although coal consumption in China surpassed 50% of the global total [3,4], it still continuously grows at a steady rate every year. Therefore, coal will remain one of the most important energy sources in China in the future. Global coal mining has a significant impact on the ecological environment, particularly the generation of industrial solid waste known as coal gangue. These wastes account for approximately 10–20% of current worldwide coal production, with a global accumulation of more than 10 billion tons. In China alone, more than 7 billion tons of coal gangue are produced each year, but the utilization rate is only 25–30%. The resulting large amounts of coal gangue are piled on the ground, encroaching on the land and bringing serious environmental risks. The excessive accumulation of coal gangue readily causes spontaneous combustion [5,6,7,8], resulting in the emission of toxic gases such as SO_2_, NO_x_, CO, CO_2_, H_2_S, and P_2_O_5_ [9,10]. These emissions not only cause serious air pollution but may also trigger natural disasters such as acid rain and mudslides. Furthermore, the weathering of coal gangue under the action of rainfall will also release heavy metals into the environment. This leaching process contaminates the soil, causing a decline in soil nutrients and quality and disrupting soil balance. Some pollutants flow into rivers and groundwater, further damaging water sources, posing a serious threat to the ecological balance, and endangering human life and property [11,12,13].

Although coal gangue has the characteristics of solid waste, it also contains certain resources. Coal gangue is primarily composed of SiO_2_, Al_2_O_3_, a small amount of Fe_2_O_3_, and trace amounts of heavy metals such as As, Hg, Pb, Zn, Cd, Cr, and Cu [14,15], in which the SiO_2_ content ranges from 30% to 65%, while the Al_2_O_3_ content is typically below 30%. However, Al_2_O_3_ levels can reach up to 40% in some high-alumina gangue. In general, the Fe_2_O_3_ content is relatively low at approximately 2–10%. Consequently, coal gangue contains rich mineral resources that can be used as industrial raw materials to produce various chemical products, including cement, coarse aggregates, bricks, ceramics, and zeolites [16]. In traditional applications, most of the coal gangue is landfilled, used to produce low-value-added chemical products, building materials, roadbeds, and foundation materials, or burned for power generation [17]. These applications greatly waste the minerals contained in gangue, resulting in a low resource utilization rate and the low-end homogeneity of downstream products. In recent years, many researchers have focused on exploring high-value applications of gangue, aiming to turn waste into treasure [18,19]. Gangue, which poses severe challenges to solid waste treatment, has become a research hotspot in the field of environmental protection and resource reuse. In some developing countries and regions, technical and financial limitations continue to hinder effective coal gangue management, making it one of the most challenging issues in global solid waste treatment [20].

Importantly, the high-value utilization of coal gangue is an inevitable requirement for current green and sustainable development and is expected to play a vital role in achieving a circular economy in the global coal industry [21]. The extraction of valuable elements from coal gangue is a crucial method of resource utilization [22,23]. For example, Shao et al. applied a thermal activation HNO_3_ method to extract useful components from coal gangue, and the leached residue obtained had a large specific Brunauer–Emmett–Teller (BET) surface area and could be used as an effective source of silica [24]. Similarly, Kong et al. used hydrochloric acid to extract iron (Fe) and aluminum (Al) from coal gangue, established a leaching kinetic model for Al and Fe ions, and explored the effects of experimental parameters on leaching outcomes [25]. Xie et al. applied a hydrothermal acid leaching method using sulfuric acid to extract lithium from gangue, achieving a lithium leaching rate of 84.42% under optimized reaction conditions. This process positions coal-based lithium as a new type of lithium energy source [26]; however, the lithium content in gangue is typically low and insufficient to meet the substantial industrial market demand. Yang et al. successfully prepared iron oxide red with a purity of 99.16% through high-temperature activation and acid leaching of high-iron gangue [27], which complies with national standards and can be utilized as a high-performance soft magnetic ferrite material in electronic technology. Additionally, studies have shown that Fe and Al ions extracted from coal gangue can be used to prepare flocculants and coagulants, such as polyaluminum chloride and polyaluminum ferric chloride [28,29], which are used in wastewater treatment. Xiao et al. used hydrochloric acid as the leaching agent to produce alumina with a purity of 98.70% by acid leaching and used the leached residue and anthracite to obtain silicon carbide with a purity of 76.1% through carbon thermal reduction [30]. This study used coal gangue as a siliceous chemical raw material to synthesize nano-silicon carbide from high-carbon waste such as waste tires and liquefied residues. This investigation is rarely reported in the literature.

Silicon carbide (SiC), as an important semiconductor material, was discovered by Acheson in 1892 and can be synthesized by the reaction of silicon dioxide and carbon. Production on a large scale was achieved the following year. Industrially produced SiC is primarily classified into two types, black SiC and green SiC, both of which are hexagonal crystals. Silicon carbide can maintain excellent physical and chemical properties under extreme conditions and has unique electronic properties such as a wide band gap and high critical breakdown field strength [31,32]. Due to the advantages of corrosion resistance, high mechanical strength, and high temperature and radiation resistance, SiC is widely used in ultra-high-temperature refractories, functional ceramics, chemical catalyst carriers, the electronics industry, semiconductors, and other fields [33,34,35,36]. Notably, the unique and exceptional nanoscale properties of nano-SiC exhibit prominent advantages in nano-sized electrical appliances, nanosensors, and other nano electronic devices. Additionally, nano-SiC is a promising candidate for applications in aerospace, high-performance compliant materials, nanofilms, and other high-tech research fields [37,38,39]. The methods reported so far for fabricating SiC mainly include carbothermal reduction, polymer precursor pyrolysis, chemical vapor deposition, gel methods, microwave heating, and discharge plasma sintering [40,41,42,43,44,45,46]. Among them, the carbothermal reduction method is highly efficient and low-cost and is currently the predominant industrial method for producing SiC, using petroleum coke and high-purity quartz sand as raw materials.

Research is increasingly focused on the use of cheaper raw materials to produce SiC. This strategy offers a viable solution for managing hazardous solid waste, promotes resource recycling, and is of great significance to environmental protection and sustainable development. In particular, large amounts of waste tires and kerosene co-refining residues have become major solid waste issues due to their inability to degrade naturally. It is estimated by 2023, the number of discarded waste tires may reach 5 billion, posing a threat to the environment comparable to that of coal gangue [47,48,49,50]. Waste tires containing a high carbon content can serve as a carbon source to produce carbon by pyrolysis and sintering. Similarly, kerosene co-refining residues with carbon contents of up to 80% [51] also have potential applications. High-carbon waste can become valuable carbon sources in the synthesis of SiC [52].

This study is dedicated to pioneering a new sustainable and affordable method of converting various wastes into valuable advanced materials such as nano-SiC. Advanced nano-SiC materials with various morphologies were synthesized using coal gangue, waste tires, and kerosene co-refining residue. The effects of feedstock, temperature, time, ingredient ratio, and reaction mechanism on the preparation of nano-SiC were investigated in depth. This study not only improves the utilization rate of solid wastes of coal gangue, waste tires, and kerosene co-refining residue but also broadens the choice of raw material sources for SiC production. Ultimately, this approach aims to mitigate the serious environmental issues caused by solid wastes worldwide, thereby promoting ecological protection and sustainable economic development.

## 2. Results and Discussion

### 2.1. Characterization of Coal Gangue, Waste Tires, and Kerosene Co-Refining Residue

For initial and calcined acid-leached coal gangue, their industrial analysis and main chemical composition are shown in Table 1 and Table 2. As can be seen in Table 1, the results of the proximate analysis show that the initial coal gangue used in this experiment contains 2.49 wt% moisture (M_ad_), 82.14 wt% ash (A_ad_), 11.14 wt% volatile matter (V_ad_), and 4.23 wt% fixed carbon (FC_ad_), pointing to a high ash content and a low organic composition. The initial coal gangue is mainly composed of 56.95% SiO_2_, 29.16% Al_2_O_3_, 4.35% Fe_2_O_3_, and a small amount of metal oxides such as K, Ca, Mg, Na, etc., implying high Si and Al contents (Table 2). After high-temperature activation and acid treatment, the ash content consisted of SiO_2_ (95.53 wt%) and alumina (2.62 wt%), which increased from 82.14 wt% to 94.9 wt%, indicating a high silica content. After hydrochloric acid leaching, alumina and a trace amount of metal oxides are removed to obtain acid-leached activated gangue residue with 95.53 wt% SiO_2_, indicating a relatively high purity. The improvement of SiO_2_ purity is attributed to the conversion of metal mineral salts into metal oxides during the high-temperature calcination process, and the reaction of metal oxide with acid during acid leaching. The high SiO_2_ content in acid-leached activated gangue residue can be further used as a silicon source for the preparation of SiC.

**Figure 1:**
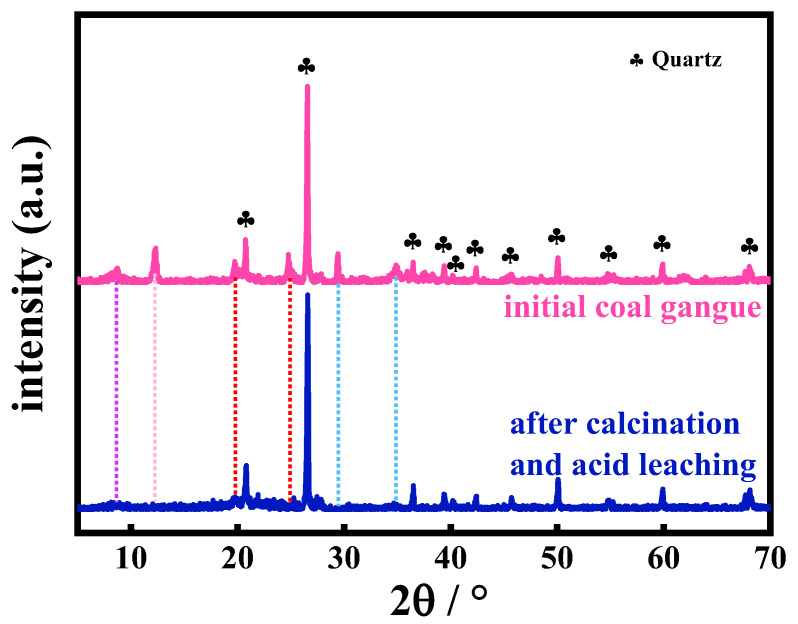
XRD patterns of the initial coal gangue and after calcination and acid leaching.

To verify the crystalline composition of the initial coal gangue, as well as after calcination and acid treatment, XRD spectra were measured. As shown in Figure 1, the XRD spectra clearly indicate that the main component of the coal gangue, both before and after acid leaching, is predominantly quartz (ICDD PDF No. 77-1060). The XRD pattern before acid leaching shows the presence of some impurities in the coal gangue. After acid leaching, the intensity of some impurity peaks weakened or disappeared. This is due to the dissolution of certain mineral phases, such as carbonates and silicates, in the coal gangue during the acid-leaching process, leading to the reduction or disappearance of the diffraction peaks of these mineral phases, which is in good agreement with the proximate analysis and chemical composition in Table 1 and Table 2.

**Figure 2:**
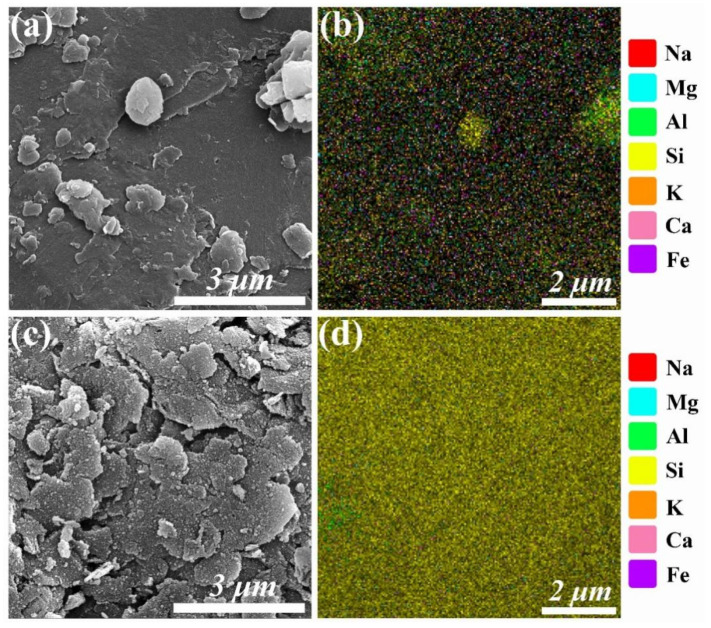
SEM image and corresponding SEM-EDS elemental mapping of (**a**,**b**) the initial coal gangue and (**c**,**d**) calcined acid-leached coal gangue residue.

Figure 2a indicates the morphology of the initial coal gangue, where no uniformly regular shapes are observed. Figure 2b exhibits the distributions of all elements in the initial coal gangue, which is in accordance with those in Table 2. After calcination and acid leaching, the calcined and acid-leached coal gangue still preserved its irregular shape (Figure 2c) and its parent, but the elemental mapping shows that silicon is dominant (Figure 2d), corroborating the substantial removal of Al and Fe metals and organic components, and indirectly confirming the importance of the acid-leaching process. This is also in good agreement with Table 2.

Figure 2 presents the EDS spectra of coal gangue before and after acid leaching, revealing a striking contrast between the two states. Prior to acid leaching, the coal gangue contained a relatively high amount of impurities, primarily in the form of metal oxides. This is evident from the EDS scan, which detected a variety of metallic elements in the raw coal gangue. However, after acid leaching, the surface of the coal gangue was dominated by silicon (Si), with only trace amounts of aluminum (Al) remaining. This observation aligns with the chemical composition analysis, which showed that the primary components of the raw coal gangue were SiO_2_ (56.95%), Al_2_O₃ (29.16%), Fe_2_O_3_ (4.35%), K_2_O (2.32%), CaO (2.04%), MgO (1.89%), and Na_2_O (1.27%). After acid leaching, the composition shifted significantly, with SiO_2_ accounting for 95.53%, while the concentrations of other oxides such as Al_2_O_3_ (2.62%), Fe_2_O_3_ (0.13%), K_2_O (0.4%), CaO (0.031%), MgO (0.039%), and Na_2_O (0.16%) were drastically reduced. 

It is important to note that the acid leaching of coal gangue is not the primary focus of our research. Instead, our goal is to utilize this method to remove the oxides present in coal gangue, thereby ensuring the successful synthesis of silicon carbide (SiC). The variations in the morphology of the synthesized SiC are hypothesized to result from the use of different carbon sources, which may influence the formation process. We believe that the synthesis of SiC involves simultaneous gas–gas and gas–solid reactions, and these reactions may be influenced by the type of carbon material used. The formation of SiC is an extremely complex and multi-step process, involving high temperatures, chemical reactions, crystal growth, and phase transformations. Although extensive research has been conducted on its surface properties and macroscopic morphology, a deep understanding of the intermediate reaction pathways, microstructural evolution, and kinetic mechanisms remains limited. Current studies are largely confined to surface-level observations, and the intrinsic formation mechanisms have yet to be fully elucidated. As a result, precise control and prediction of the SiC synthesis process remain significant challenges. Further advancements in this field will require interdisciplinary collaboration and the application of advanced characterization techniques and theoretical simulations to gradually unravel the complexities of this process.

**Figure 3:**
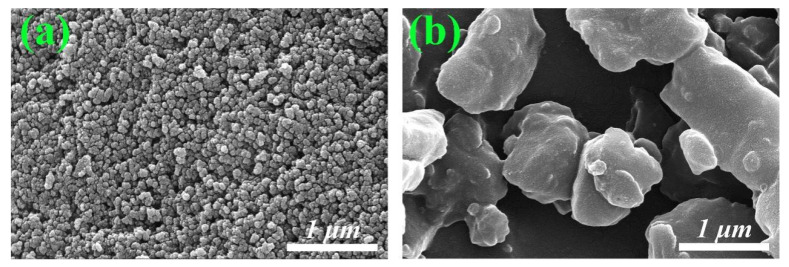
SEM images of (**a**) the carbonized waste tires and (**b**) the pre-treated kerosene co-refining residue.

Similarly, for the carbonized waste tire residue and the pre-treated kerosene co-refining residue, their compositions from the proximate analysis and elemental analysis are listed in Table 3 and Table 4, respectively. Interestingly, the carbon contents are very similar to each other and both can be used as a carbon source for further utilization. In comparison, the morphology of both the carbonized waste tire residue and the pre-treated kerosene co-refining residue is completely different (Figure 3). As shown in Figure 3a, the carbonized waste tires are composed of a dense aggregation of nano-sized spherical particles, while the pre-treated kerosene co-refining residue primarily consists of irregular bulky lumps (Figure 3b).

**Figure 4:**
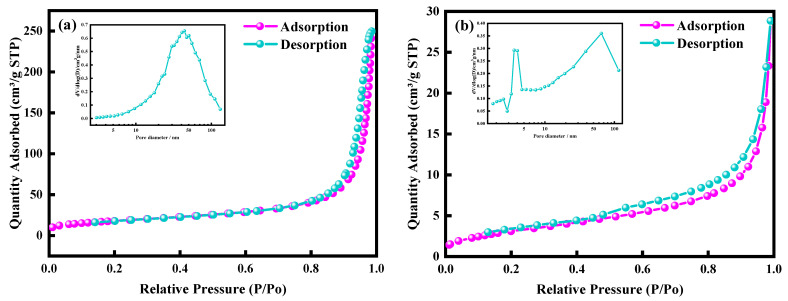
N_2_ adsorption isotherms and pore size distribution of the of carbonized waste tires (**a**) and pre-treated kerosene co-refining residue (**b**).

As a carbon source for preparing SiC, the difference in morphology probably impacts the shapes and properties of the final nanostructured SiC. The specific surface area and pore structure properties of carbon materials are among the factors contributing to the formation of different morphologies of silicon carbide. Figure 4 shows the N_2_ adsorption isotherms of two carbon material samples. According to the IUPAC classification, both carbon materials exhibit type IV isotherms and H3-type hysteresis loops, indicating the presence of mesoporous structures. As can be observed from Figure 4, the pore size distribution of the carbonized waste tires is mainly in the range of 10–50 nm. In contrast, the pre-treated kerosene co-refining residue has relatively smaller pore sizes, which are predominantly around 10 nm. Table 5 reveals that the specific surface area of the carbonized waste tires reaches 62.07 m^2^/g, with an average pore size of 27.75 nm. On the other hand, the specific surface area of the pre-treated kerosene co-refining residue is relatively low at only 11.52 m^2^/g, and the average pore size is 12.59 nm. A high specific surface area implies that the carbonized waste tires possess a greater number of active sites. This is conducive to the formation of more nucleation sites during high-temperature reactions, potentially facilitating the uniform growth of nanofibers. Additionally, the large pore sizes are beneficial for the diffusion of reactants and products. For example, they facilitate the diffusion of silicon source gas reactants and the expulsion of products, thereby contributing to the formation of a uniform nanofiber structure. Conversely, a small specific surface area may restrict the diffusion of reactants and the expulsion of products. This can lead to non-uniform reactions, affecting the formation of nanofibers. Instead, it favors the formation of particulate or irregular block/flake-shaped silicon carbide with non-uniform morphologies and rough surfaces.

### 2.2. Synthesis and Characterization of SiC

**Figure 5:**
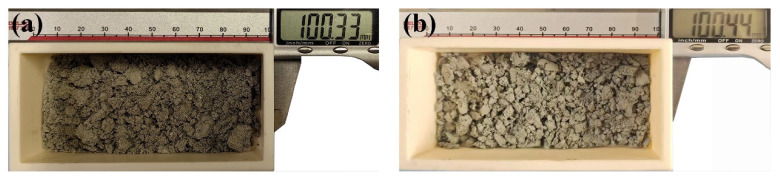
(**a**) SiC (1) prepared by the reaction of the calcined acid-leached gangue with carbonized waste tires. (**b**) SiC (2) derived from the reaction of the calcined acid-leached gangue with the pre-treated kerosene co-refining residue.

The calcined acid-leached gangue was thoroughly mixed with the carbonized waste tires and the kerosene co-refining residue to form a “homogeneous” mixture, and then thermally treated at 1600 °C for 4 h under Ar protection to yield the dark green products SiC (1) and SiC (2), respectively. Their masses are 5.35 g and 4.94 g, respectively. The color has likely been influenced by heteroatomic doping, such as N and Al in crystalline SiC. Figure 5a shows the SiC (1) product prepared by the reaction of the calcined acid-leached gangue with the carbonized waste tires, and Figure 5b indicates that SiC (2) stemmed from the thermal reaction of the calcined acid-leached gangue with the pre-treated kerosene co-refining residue.

The initial mass of the as-prepared SiC (1) was 5.35 g. Due to the high temperature inside the furnace during the cooling process after sintering, the surface of the silicon carbide was oxidized, resulting in the formation of a small amount of SiO_2_. Therefore, the obtained powder was immersed in a beaker containing a 20% HCl aqueous solution for 12 h to remove impurities. After drying at 70 °C for 12 h, it was transferred to a polytetrafluoroethylene cup and treated with a 45% HF aqueous solution in a fume hood for 12 h. Following filtration, the solid was dried at 70 °C for 12 h, yielding 4.94 g of silicon carbide. Preliminary calculations indicated a silicon carbide content of 92.3%. The obtained solid silicon carbide was subjected to the same acid-leaching and impurity removal steps again, ultimately yielding 4.90 g of silicon carbide. The calculated purity of the silicon carbide was 99.19%. After undergoing the same treatment steps, SiC (2) achieved a purity of 98.23%.

**Figure 6:**
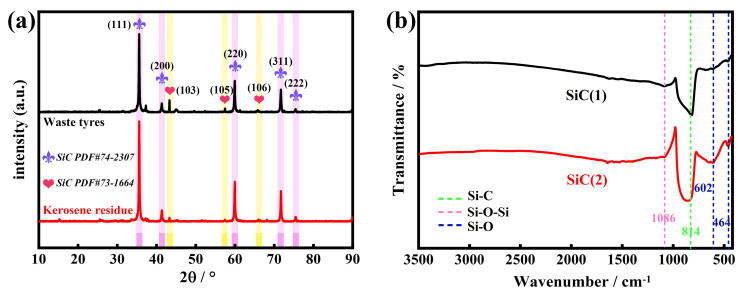
(**a**) XRD patterns and (**b**) FT-IR spectra of SiC (1) and (2).

**Figure 7:**
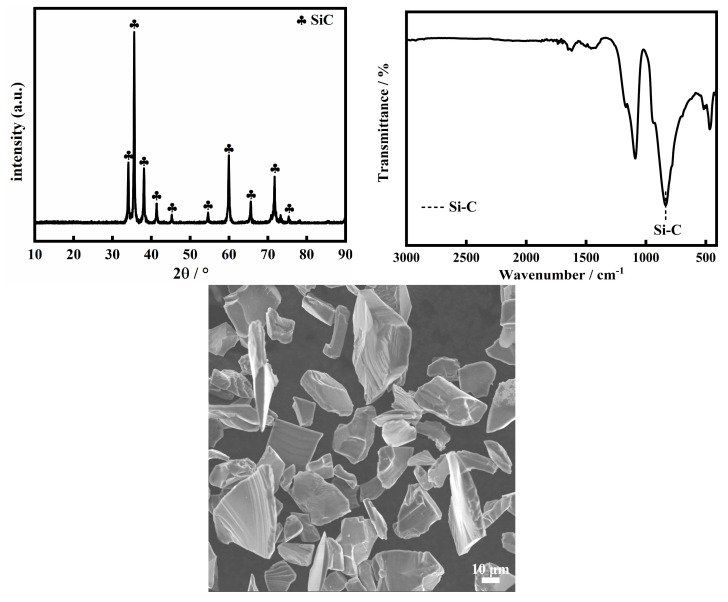
XRD patterns, FT-IR spectra, and SEM of commercial-grade SiC.

As can be seen in Figure 6a, SiC (1) and (2) show a similar XRD pattern, although they stemmed from different carbon precursors. The diffraction peaks at 2θ = 35.65°, 41.37°, 59.98°, 71.73°, and 75.42° are indexed as the (111), (200), (220), (311), and (222) planes of the cubic crystal system 3C-SiC (β-SiC), and the peaks at 2θ = 43.3°, 57.46°, and 65.8°, belong to the (103), (105), and (106) planes of the hexagonal crystal system 4H-SiC (α-SiC). In addition, the weak diffraction peaks assigned to SiO_2_ are also observed at 2θ = 26°, 19.1°, and 15.22°. The results confirm the co-existence of α, β-SiC and a trace amount of SiO_2_ in SiC (1) and (2). To further elucidate the compositions, the Fourier-transform infrared resonance (FT-IR) spectra of SiC (1) and (2) were measured and are shown in Figure 6b. The characteristic vibration bands at 1086, 814, 602, and 464 cm^−1^ are observed, in which the stronger absorption band at 814 cm^−1^ is attributed to the stretching mode of Si-C in SiC [53], while the bands with a weak intensity at 1086 cm^−1^ and 602 and 464 cm^−1^ correspond to the anti-symmetric stretching mode of Si-O-Si and the bending vibration mode of Si-O [54], respectively, further indicating the presence of a trace amount of SiO_2_ in the SiC. The existence of a trace amount of SiO_2_ originates from the formation of a protective SiO_2_ film on the SiC surface at high temperatures [55], or the incomplete removal of unreacted SiO_2_ during HF immersion. In this regard, we compared the XRD and FTIR spectra of commercial-grade silicon carbide and found that traces of SiO_2_ could also be observed in the spectra in Figure 7. Therefore, we believe that the silicon carbide we synthesized matches the purity of commercial-grade silicon carbide.

**Figure 8:**
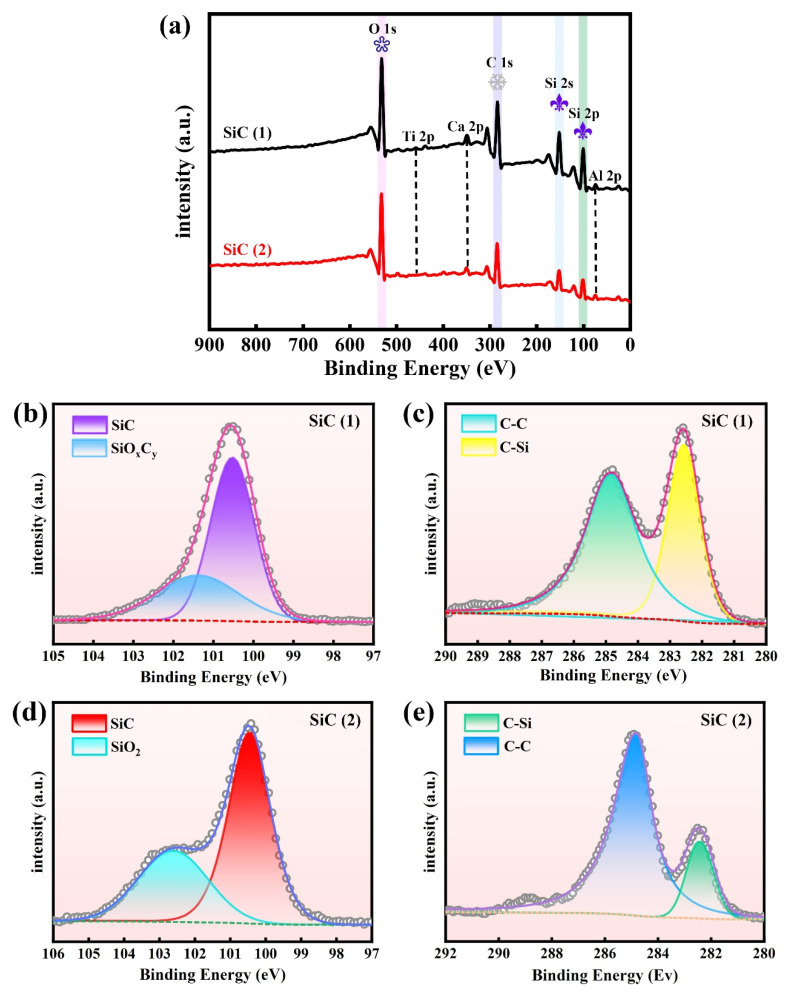
(**a**) XPS survey scan spectra of SiC (1) and (2). High-resolution (**b**) Si 2p and (**c**) C 1s XPS spectra in SiC (1), and (**d**) Si 2p and (**e**) C 1s in SiC (2).

To further investigate the valence state of the elements and the surface chemical composition of SiC (1) and (2), XPS spectra were collected. As shown in Figure 8a, the XPS survey scan spectra of SiC (1) and (2) clearly verify the presence of dominant Si, C, and O, as well as the existence of a trace amount of the metals Ca, Al, and Ti, in SiC. A strong O 1s peak likely stems from the residual SiO_2_ or other metal oxides such as CaO, Al_2_O_3_, and TiO_2_ in SiC, which is in accordance with the Ti 2p, Ca 2p, and Al 2p signals observed in Figure 8a. As shown in Figure 8b, high-resolution Si 2p XPS is fitted into a prominent peak at 100.46 eV and a weak peak at 101.3 eV, in which the former can be attributed to the binding energy of Si in the Si-C bond in SiC (1) and the latter is the binding energy of Si in the trace amount of SiO_2_ in SiC (1) [56]. The two C 1s peaks at 282.32 eV and 284.55 eV correspond to the C-Si and C-C bonds in SiC (1), respectively (Figure 8c). For SiC (2), high-resolution Si 2p and C 1s XPS spectra (Figure 8d,e) show similar results to those of SiC (1), with only the binding energies possessing differences. These results further corroborate the successful synthesis of SiC, with a trace amount of the dopant SiO_2_.

**Figure 9:**
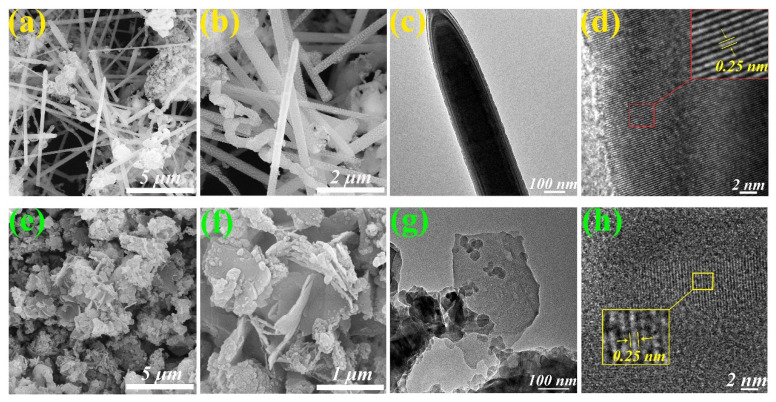
(**a**,**b**) SEM and (**c**,**d**) (high-resolution) TEM images of SiC (1). (**e**,**f**) SEM and (**g**,**h**) (high-resolution) TEM images of SiC (2).

The morphology of SiC (1) and (2) was characterized by an SEM microscope. Figure 9a indicates the morphology of SiC (1), a dominantly characteristic nanofiber with a length of about 5–10 μm and a few bulky aggregations (SiC) due to different crystalline phases (α, β-SiC). As shown in Figure 9b, the surface is relatively smooth when observed at high magnification, but with a limited number of distorted whiskers and SiC nanoparticles. This phenomenon is probably attributed to the influence of temperature gradients and atmospheric conditions during high-temperature processing, which leads to uneven growth rates in different crystal plane directions. Additionally, the presence of internal stresses and defects may cause crystal deformation, resulting in a twisted or spherical particle structure. The diameter of a single fibrous SiC is about 200 nm (Figure 9c), and lattice fringes with a lattice spacing of 0. 25 nm belonging to the cubic or hexagonal crystal system are readily observed everywhere (Figure 9d), indicating a characteristic crystalline structure of SiC. The results are in accordance with the XRD analysis.

**Figure 10:**
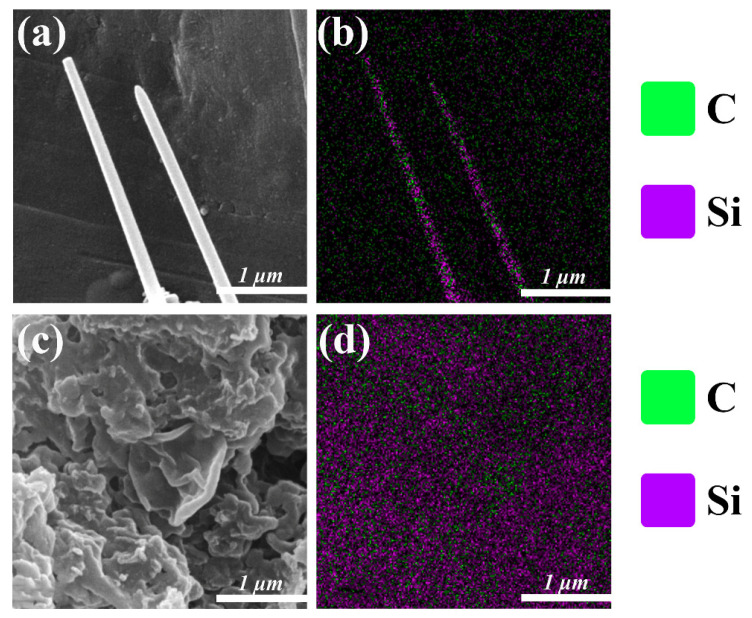
(**a**) SEM image for measuring (**b**) EDS elemental mapping of C and Si in SiC (1). (**c**) SEM image for measuring (**d**) EDS elemental mapping of C and Si in SiC (2).

Interestingly, the SEM image of SiC (2) presented in Figure 9e indicated an irregular morphology consisting of blocks and flakes. The surface of nanosheet-like SiC is relatively smooth and flat (Figure 9f). Block-like aggregations with diameters in the range of approximately 20~30 nm on the SiC nanosheet are likely amorphous carbon particles (Figure 9g), while lattice fringes with a spacing of 0.25 nm are visible (Figure 9h), implying the co-existence of a crystalline structure of SiC (2) and amorphous carbon particles. In addition, SEM-EDS elemental mappings of SiC (1) and (2) also clearly indicate the uniform distribution of C and Si in whole nanostructured SiC (Figure 10).

#### 2.2.1. Effect of Temperature on the Preparation of Silicon Carbide

**Figure 11:**
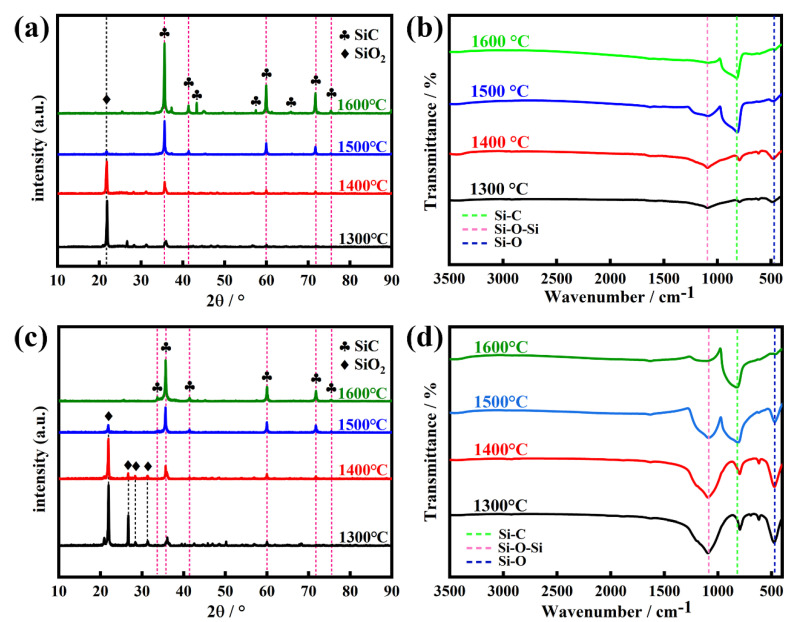
(**a**) XRD patterns and (**b**) FT-IR spectra of the products prepared by the reaction of the calcined coal gangue and the carbonized waste tire residue at various reaction temperatures. (**c**) XRD patterns and (**d**) FT-IR spectra of the products prepared by the reaction of the calcined coal gangue and the pre-treated kerosene co-refining residue at different reaction temperatures.

Figure 11 shows the XRD patterns and FT-IR spectra of SiC synthesized from calcined coal gangue with carbonized waste tires and with pre-treated kerosene co-refining residue at various reaction temperatures. As shown in Figure 11a,c, the XRD patterns show a strong characteristic diffraction peak at 2θ = 21.94°, belonging to SiO_2_, and a weak characteristic diffraction peak at 2θ = 35.65°, assignable to SiC at 1300 °C and 1400 °C and pointing to a composition that is predominantly SiO_2_ and has a low amount of SiC in the final product. However, the XRD patterns of the products preprepared by two different precursors at 1500 °C indicate a remarkable increase in the intensity of the diffraction peak at 2θ = 35.65° for SiC and a significant decrease in the intensity of the diffraction peak at 2θ = 21.94° for SiO_2_, while some new characteristic diffraction peaks appear at 2θ = 41.37°, 59.98°, 71.73°, and 75.42°, which are attributed to SiC, implying that SiC increases with increasing reaction temperatures in final products. This result hints that the improvement of the reaction temperature is conducive to the formation of SiC or the conversion of SiO_2_ into SiC in the presence of carbon. It is worth noting that in the products fabricated at 1600 °C, the characteristic diffraction peaks assignable to SiO_2_ completely disappeared, while only the diffraction peaks of SiC are observed, indicating a characteristic SiC product and further demonstrating the crucial role of a high reaction temperature for the preparation of SiC.

As evidence, with increasing reaction temperatures from 1300 °C to 1600 °C, the FTIR spectra of the obtained products (Figure 11b,d) exhibit that the signals of vibration bands at 1086, 602, and 464 cm^−1^, attributable to Si-O-Si and Si-O bonds from SiO_2_, gradually weakened until they completely disappeared, while the signal of Si-C vibration from SiC remarkably increased until only the Si-C band was a unique vibration in the high-purity SiC product synthesized at 1600 °C, directly corroborating that pure SiC stems from the high-temperature reaction of the calcined coal gangue residue with the carbonized waste tires or the pre-treated kerosene co-refining residue at a temperature not less than 1600 °C.

#### 2.2.2. Effect of Reaction Time on the Preparation of Silicon Carbide

**Figure 12:**
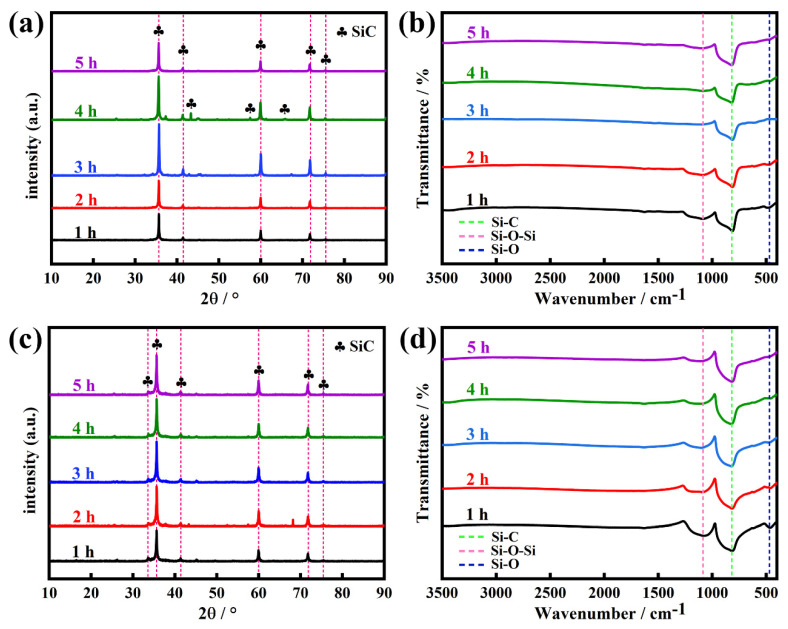
(**a**) XRD patterns and (**b**) FT-IR spectra of SiC prepared through the reaction of the calcined coal gangue with the carbonized waste tires at 1600 °C for expected reaction times from 1 h to 5 h. (**c**) XRD patterns and (**d**) FT-IR spectra of SiC prepared through the reaction of the calcined coal gangue with the pre-treated kerosene co-refining residue at 1600 °C for expected reaction times from 1 h to 5 h.

We investigated the effect of reaction time on the quality of SiC prepared by the reaction of the calcined coal gangue with the carbonized waste tires and with the pre-treated kerosene co-refining residue at 1600 °C. Figure 12 shows the XRD patterns and FT-IR spectra of SiC synthesized from the calcined gangue with the carbonized waste tire residue and with the pre-treated kerosene co-refining residue for various reaction times from 1 to 5 h. The results from the XRD patterns (Figure 12a,c) show that the alteration of the reaction time did not have a significant effect on the quality of SiC, regardless of whether the SiC was derived from the reaction of the calcined coal gangue with the carbonized waste tires or with the pre-treated kerosene co-refining residue. However, the results from the FT-IR spectra (Figure 12b,d) show that a long reaction time is very beneficial for the complete conversion of SiO_2_ into SiC in the presence of a carbon precursor. When the reaction time changed from 1 h to 2 h, the obtained SiC still contained a trace amount of unreacted SiO_2_. With an extension of the reaction time from 3 h to 5 h, the obtained SiC is a high-purity product without any SiO_2_; hence, the optimal reaction time is 4 h.

#### 2.2.3. Effect of the Ratio of the Calcined Gangue to the Carbonized Waste Tires or the Pre-Treated Kerosene Co-Refining Residue

**Figure 13:**
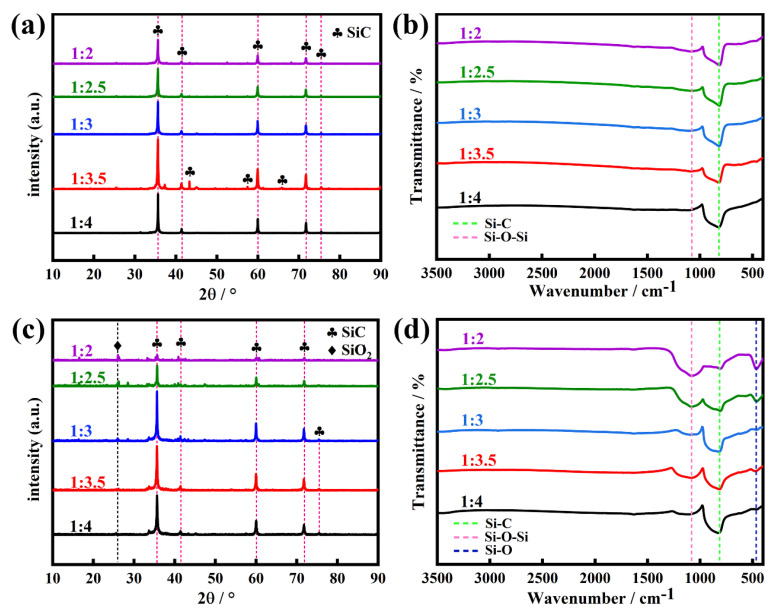
XRD patterns and FT-IR spectra of as-made SiC prepared from (**a**,**b**) the calcined gangue with the carbonized waste tires, and (**c**,**d**) the calcined gangue with the pre-treated kerosene co-refining residue by adjusting mass ratios from 1:2 to 1:4.

To evaluate the effect of the ratio of starting materials on the quality of SiC, we adjusted the molar ratio of the calcined gangue to the carbonized waste tires or the pre-treated kerosene co-refining residue from 1:2 to 1:4 while keeping other conditions identical (1600 °C, 4 h). Figure 13 shows the XRD patterns and FT-IR spectra of SiC synthesized from the calcined gangue with the carbonized waste tires and with the pre-treated kerosene co-refining residue with various mass ratios. When the mass ratio of the calcined gangue with the carbonized waste tires was adjusted from 1:2 to 1:4, the XRD patterns and FTIR spectra of the as-made SiC did not show a significant SiO_2_ content (Figure 13a,b), verifying the absence of SiO_2_ in SiC and confirming the high purity of as-made SiC. However, the XRD patterns (Figure 13c) of SiC prepared from the calcined gangue and the pre-treated kerosene co-refining residue showed the presence of the characteristic diffraction peaks of SiO_2_ at 2θ = 26° when the molar ratios changed from 1:2 and 1:3.0. This reason is probably attributed to the fact that the effective carbon produced by the high-temperature carbonization of the pre-treated kerosene co-refining residue is insufficient to meet the amount of carbon required to convert silicon dioxide into silicon carbide; therefore, a trace amount of SiO_2_ still exists in SiC. The FT-IR spectra (Figure 13d) also exhibited the clear co-existence of Si-O-Si and Si-O vibrations at 1086 and 464 cm^−1^ from SiO_2_ and a Si-C bond at 814 cm^−1^ in SiC, suggesting the existence of SiO_2_ in SiC. Note that by increasing the amount of the pre-treated kerosene co-refining residue (1:3.5), the SiO_2_ content in SiC remarkably decreased. When the mass ratio of the calcined gangue and the pre-treated kerosene co-refining residue was 1:4, the SiO_2_ phase completely disappeared and only the SiC phase was detected, which was confirmed by the XRD pattern and IR spectrum (Figure 13c,d).

### 2.3. Mechanistic Study of Silicon Carbide Synthesis

The general reaction equation for the preparation of SiC through the carbothermal reduction of SiO_2_ with C in a Si-C-O system is as follows:

(1) $$SiO_{2}+3C=\mathrm{SiC}+2\mathrm{CO}$$

However, the arrangement of SiC is often influenced by several intermediate reactions in this specific process, which may include the following reactions:

(2) $$\mathrm{Si}O_{2}+C=\mathrm{SiO}\left(g\right)+\mathrm{CO}\left(g\right)$$

(3) $$\mathrm{SiO}\left(g\right)+\mathrm{CO}\left(g\right)=\mathrm{SiO}\left(g\right)+CO_{2}\left(g\right)$$

(4) $$CO_{2}\left(g\right)+C=2\mathrm{CO}\left(g\right)$$

(5) $$\mathrm{SiO}\left(g\right)+2C=\mathrm{SiC}+\mathrm{CO}\left(g\right)$$

(6) $$2\mathrm{SiO}\left(g\right)+3C=2\mathrm{SiC}+CO_{2}\left(g\right)$$

(7) $$\mathrm{SiO}\left(g\right)+3\mathrm{CO}=\mathrm{SiC}+2CO_{2}\left(g\right)$$

(8) $$3\mathrm{SiO}\left(g\right)+\mathrm{CO}\left(g\right)=\mathrm{SiC}+2\mathrm{Si}O_{2}$$

The Gibbs free energy and entropy increase principle is used to define the standard Gibbs free energy change (Δ_r_$G_{m}^{\Theta }$). Therefore, at a specific temperature and pressure, the change in the Gibbs free energy of a reaction determines both the direction and extent of the chemical reaction. The Gibbs free energy can be calculated using the following equations:

(9) $$\Delta_{r}H_{m}^{\Theta }\left(P,T\right)=\sum \Delta_{f}H_{m}^{\Theta }{\left(P,T\right)}_{\mathrm{resultant}}\;-\sum \Delta_{f}H_{m}^{\Theta }{\left(P,T\right)}_{\mathrm{reactant}}$$

(10) $$\Delta_{r}S_{m}^{\Theta }\left(P,T\right)=\sum \Delta_{f}S_{m}^{\Theta }{\left(P,T\right)}_{\mathrm{resultant}}\;-\sum \Delta_{f}S_{m}^{\Theta }{\left(P,T\right)}_{\mathrm{reactant}}$$

(11) $$\Delta_{r}G_{m}^{\Theta }\left(P,T\right)=\Delta_{r}H_{m}^{\Theta }\left(P,T\right)-T\Delta_{r}S_{m}^{\Theta }(P,T)\;$$

The values of Δ_r_$G_{m}^{\Theta }$ for Equations (1)–(8) at various temperatures were obtained by consulting a thermodynamic database and performing software calculations.

**Figure 14:**
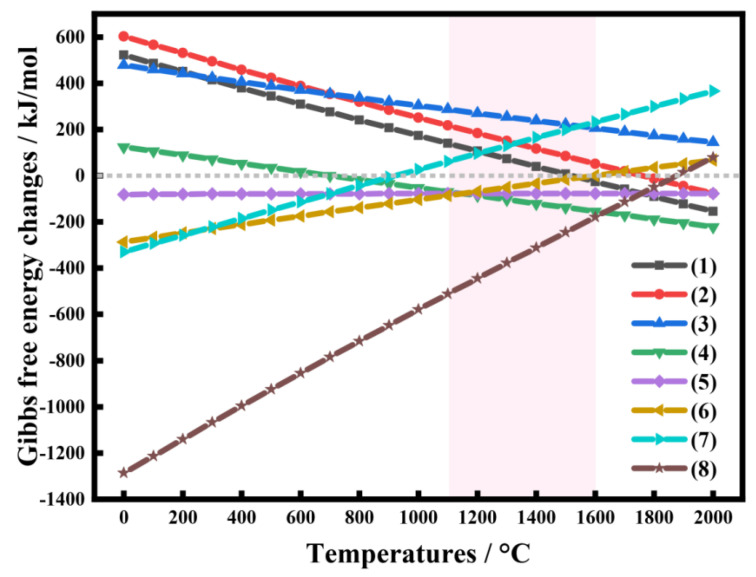
Temperature-dependent Gibbs free energy changes for the preparation of silicon carbide.

According to Figure 14, Reaction (1) can theoretically proceed spontaneously at temperatures higher than 1530 °C. This indicates that the carbothermal reduction for the preparation of SiC is feasible at 1600 °C in this study. The standard Gibbs free energy change (Δ_r_$G_{m}^{\Theta }$) of Reactions (2) and (3) decreases with increasing temperatures, suggesting that SiO and CO primarily originate from these two reactions. In fact, the growth of SiC can be enhanced by optimizing the reaction conditions, thereby driving the reaction system to favor Reactions (2) and (3), producing more SiO intermediates.

SiC is primarily produced through Reactions (5)–(8). As illustrated in Figure 14, the standard Gibbs free energy change (Δ_r_$G_{m}^{\Theta }$) is less than 0 for Reactions (5), (6), and (8) at temperatures below 1600 °C, indicating that these reactions can theoretically occur spontaneously. Conversely, for Reaction (7), Δ_r_$G_{m}^{\Theta }$ is greater than 0 at temperatures above 1000 °C, suggesting that this reaction cannot proceed spontaneously. Therefore, SiC can ultimately be synthesized from Reactions (5), (6), and (8), in which SiO vapor reacts with CO gas and solid C to produce SiC.

**Figure 15:**
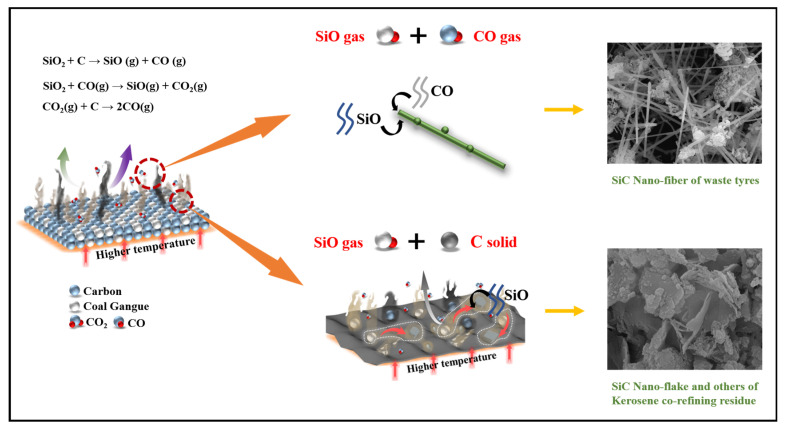
Diagram of the growth mechanism of SiC.

Based on these reactions and their thermodynamic properties, we proposed a growth model for SiC, as shown in Figure 15. It identified two primary mechanisms: a gas–gas homogeneous reaction that generates nanofibrous SiC within the Si-C-O system, and a gas–solid non-homogeneous reaction that produces irregular SiC forms, such as nanoblocks and nanosheets. In the gas–gas homogeneous reaction, the intermediate SiO produced by SiO_2_ aligns with CO in the system to form SiC nuclei, thereby promoting whisker growth [57]. Conversely, in the gas–solid non-homogeneous reaction, SiO gas deposits on the surface of the carbon material, facilitating SiC growth to form nanosheets or nanoblocks. It is crucial to note that the gas–solid non-homogeneous reaction is primarily influenced by the lattice defects, pores, surface adsorption, and active sites present on the carbon material and by the partial pressure of each gas in the system. Consequently, this study used various solid wastes with high carbon contents to produce SiC with distinct morphologies. The differences in the morphology, structure, and properties of the carbon materials play a pivotal role in this process. For example, the fixed carbon content in the carbonized waste tires was higher than in the pre-treated kerosene co-refining residue, and the carbonized waste tires had a larger specific surface area and greater reactivity. This increased reactivity can lead to the generation of more CO within the system to bind the SiO and produce more SiC from the carbonized waste tires, ultimately resulting in a higher yield of nanofibrous SiC. In contrast, the carbon in the pre-treated kerosene co-refining residue was less active than in the carbonized waste tires, causing a significant amount of SiO gas to deposit on the surface of the carbon material and form various SiC structures.

SiC is renowned for its exceptional thermal stability, mechanical strength, and chemical inertness, making it an ideal material for use in high-temperature and corrosive environments. The SiC nanofibers and nanosheets developed in this study can be used in thermal protection systems and as catalyst supports.

In the current study, we are in the preliminary exploration stage of synthesizing silicon carbide with different morphologies. Based on our experimental observations, we have inferred that one of the factors influencing the formation of nanofibers and nanosheets is the difference in carbon precursors used during the synthesis process. However, we fully recognize that other factors, such as reaction conditions (temperature, pressure, and reaction time) and catalyst effects, may also play significant roles in determining the nanostructure’s aspect ratio, dimensions, and size. In our future studies, we will focus on systematically exploring the key parameters that control the nanostructure’s morphology and size, with the aim of providing a more comprehensive understanding of the synthesis mechanism.

## 3. Experimental Section

### 3.1. Materials

The coal gangue came from northern Shaanxi Province in China and 80-mesh waste tire particles from the Shaanxi Hongrui Rubber Products Company (Baoji, China). The kerosene co-refining residue came from northern Shaanxi Province in China. Hydrochloric acid (36.0~38.0%) from Sinopharm Chemical Reagent Co., Ltd. (Nanjing, China) and hydrofluoric acid (40%) from Shanghai Macklin Biochemical Technology Co., Ltd. (Shanghai, China) were purchased. Deionized water (18 MΩ) was used as a solvent.

### 3.2. Experimental Procedure

#### 3.2.1. The Pre-Treatment of Coal Gangue, Waste Tire Particles, and Kerosene Co-Refining Residue

Coal gangue was crushed into small pieces using a jaw crusher. A portion of the crushed material was then pulverized in a mill for approximately 30 s to obtain a fine powder. The resulting powder was sifted with an 80-mesh sieve. The sieved coal gangue particles were then dried in an oven at 80 °C for 24 h. After drying, an appropriate amount of gangue was uniformly placed in a corundum porcelain boat and calcined in a muffle furnace. The furnace temperature was increased by 5 °C/min to 800 °C and maintained at this temperature for 2 h to obtain calcined gangue powder. Afterwards, the calcined gangue powder was put into a 150 mL round-bottom flask and 20 wt% HCl aqueous solution was added to perform acid leaching in an oil bath under stirring. Due to the various physicochemical properties of the gangue from different origins, the acid leaching conditions also varied. In this experiment, 50 mL of 20 wt% HCl aqueous solution was used to treat 10 g of calcined gangue. The acid leaching experiment was performed at 120°C for 24 h under stirring. In this case, the removal efficiency of alumina reached 93.16%. The residues after acid leaching were collected by filtration with a Brinell funnel and dried at 80 °C for 24 h to obtain calcined acid-leached gangue residue.

In addition, 80-mesh waste tire particles from the Shaanxi Hongrui Rubber Products Company were processed to enhance the efficiency and environmental sustainability of waste tire recycling. This process significantly reduced the volume of waste tires and eliminated harmful substances. The waste tire particles were placed in a vacuum high-temperature tubular furnace (BTF-1700-S). The furnace temperature was increased by 10 °C/min to 700 °C. Under atmospheric pressure and with Ar protection and a gas flow rate of 100 mL/min, this temperature was maintained for 20 min to carbonize the tire particles. The carbonized tire particles were then ground into powder and collected, denoted as carbonized waste tire residue.

Similarly, the pre-treatment of the kerosene co-refining residue was similar to that of coal gangue. The kerosene co-refining residue was crushed into small pieces using a jaw crusher. A portion of the crushed material was then pulverized in a mill for approximately 30 s to obtain a fine powder. The resulting powder was sifted with an 80-mesh sieve. The sieved pre-treatment kerosene co-refining residue was then dried in an oven at 80 °C for 24 h. The obtained residue was marked as pre-treated kerosene co-refining residue.

#### 3.2.2. The Preparation of SiC Nanoparticles from the Calcined Acid-Leached Coal Gangue, the Carbonized Waste Tires, and the Pre-Treated Kerosene Co-Refining Residue

The calcined acid-leached gangue residue was mixed stoichiometrically with the carbonized waste tire residue and the pre-treated kerosene co-refining residue, respectively. The mixture was then placed in a planetary ball mill for 5 min. After being uniformly mixed, the “homogeneous” mixture was spread in a corundum ceramic boat with a lid and transferred into a high-temperature tube furnace to carbonize at different temperatures (1600 °C, 1500 °C, 1400 °C, and 1300 °C) under Ar protection (Ar gas flow rate was 50 mL/min) for an expected time. For example, the furnace temperature was increased by 5 °C/min to 200 °C, then by 10 °C/min to 1550 °C, and finally by 5 °C/min to 1600 °C. The furnace was then naturally cooled down to room temperature to obtain a nano-SiC product, which was further heated up to 700 °C with a heating rate of 5 °C/min in a muffle furnace and kept at this temperature for 3 h under Ar protection to afford a powdery product. The resulting powder was immersed in a 20% HCl aqueous solution in a beaker for 12 h to remove impurities. After filtration, the solid was subsequently placed in a polytetrafluoroethylene cup and treated with a 45% HF aqueous solution for another 12 h in a fume hood. The solid was collected by filtration and washing with water in a fume hood and dried at 70 °C for 12 h to obtain SiC. Finally, the purity of silicon carbide is preliminarily determined by using the mass ratio before and after acid leaching and impurity removal, thereby ascertaining the feasibility of synthesizing silicon carbide using this method.

**Figure 16:**
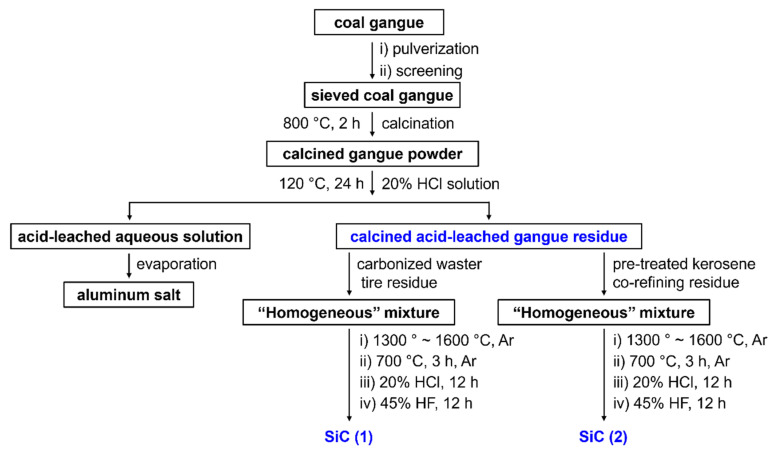
A schematic illustration of the preparation of SiC from coal gangue and waste tires or kerosene co-refining residue.

A schematic illustration of the whole process of the preparation of SiC from coal gangue with waste tires or kerosene co-refining residue is shown in Figure 16. In addition, it is worth noting that the sintered product appeared in the form of a fluffy powder, which is not necessary to further grind or specially treat. The acid-leached solution collected can also be further treated to afford metal chlorides (no further detailed discussion in this work).

### 3.3. Characterization

Elemental analyses (C, H, O, N, and S) were performed on an elemental analyzer (elementar vario Micro cube, Elementar, Langenselbold, Germany). Powder X-ray diffraction (XRD) patterns were collected on a Shimadzu XRD-6100 X-ray diffractometer (Shimadzu, Kyoto, Japan) using monochromatic Cu Kα radiation (λ = 1.5406 Å in the 2θ range of 5–100° with a scan speed of 5° per minute, and the phases were identified using the ICDD PDF database. X-ray fluorescence spectroscopy (XRF) was recorded on a wavelength dispersive X-ray fluorescence (WDXRF) instrument from Zetium, Panaco, The Netherlands. The light source was a rhodium (Rh) tube with a power of 4 kW, and a power of 2.4 kW was applied during measurements. Fourier-transform infrared (FT-IR) spectra were measured on a Nicolet iS 5 FT-IR spectrometer in the range of 4000 to 400 cm^−1^ with a KBr tablet. X-ray photoelectron spectroscopy (XPS) was conducted with a Thermo Fisher ESCALAB 250Xi spectrometer (Thermo Fisher Scientific, Waltham, MA, USA) with Al Kα radiation (hν = 1486.8 eV) as the excitation source at an operating voltage of 12.5 kV and a filament current of 16 mA. Scanning electron microscope (SEM) images were obtained on a field emission scanning electron microscope (FEI Quattro S) equipped with an EDAX ELECT PIUS energy spectrometer for mapping (Thermo Fisher Scientific, Waltham, MA, USA), which was used for the analysis. A small amount of the powdered sample was placed in a GVC-2000 ion sputtering apparatus (Gatan, Inc., Pleasanton, CA, USA) for approximately 120 s to apply a gold coating. Transmission electron microscope (TEM) images were measured on an FEI, Tecnai F20 at an operating voltage of 200 kV. A small amount of the powder was ultrasonically dispersed in anhydrous ethanol, and one to two drops of the dispersion were dripped on a copper grid and dried in air for TEM measurements.

## 4. Conclusions

In this study, solid wastes including coal gangue, waste tires, and kerosene co-refining residue were efficiently converted into useful raw materials for preparing the high-value-added product silicon carbide (SiC), thereby achieving “turning trash into treasure”. The calcined coal gangue stemmed from the initial coal gangue, and the carbonized waste tires from waste tires and the pre-treated kerosene co-refining residue were used as a silicon source and carbon source, respectively. The differences in the carbon source result in the formation of SiC with distinct morphologies—nanofibers and nanosheets—through carbothermal reduction. The effects of the reaction temperatures and times, as well as the mass ratio, on the purity and morphology of as-made SiC were investigated in detail to unveil the origin of the formation of pure SiC and optimize synthetic conditions. In addition, the calculated Gibbs free energies of the reactions also provide a primarily theoretical basis for understanding the formation of SiC. Finally, two growth mechanisms of SiC, namely the reaction of intermediate SiO with CO forming SiC-nuclei-driven nanofibrous SiC and SiO-deposited carbon surface-fabricated nuclei-induced polymorphic SiC (dominant nanosheets), are proposed. This study provides a constructive strategy to achieve the effective utilization of solid wastes and reduce the pollution of solid wastes in the environment, ultimately contributing to the sustainable development of solid waste resources. While the current study focuses on the synthesis and characterization of SiC nanofibers and nanosheets, there are also some limitations. For instance, the complex composition of the raw materials significantly affects the controllability of the morphology of the SiC products. Additionally, the carbothermal reduction process is highly temperature-dependent and often requires prolonged reaction times at high temperatures (typically above 1600 °C). This results in high energy consumption and increased production costs. In the future, we plan to explore their practical applications in the aforementioned fields in future work. Specifically, we aim to investigate their performance in “lithium-ion batteries” or “catalyst supports” and optimize their properties for targeted applications.

The synthesis of SiC via carbothermal reduction using waste tires, coal oil residue, and coal gangue offers a sustainable and economically viable approach to producing high-value materials from waste. However, challenges related to impurities, reaction efficiency, morphology control, and environmental impact must be addressed to fully realize the potential of this method. Future research should focus on optimizing raw material pre-treatment, reaction conditions, and process monitoring while exploring novel applications for the synthesized SiC. By addressing these limitations, this approach can contribute to the development of a circular economy and sustainable material production.

## Figures and Tables

**Table 1:** Proximate analysis of the initial coal gangue and after calcination and acid leaching.

Samples	M_ad_/wt%	A_ad_/wt%	V_ad_/wt%	FC_ad_/wt%
Initial	2.49	82.14	11.14	4.23
After	2.16	94.90	2.91	0.03

**Table 2:** Chemical composition of the initial coal gangue and after calcination and acid leaching.

Samples	SiO_2_ /wt%	Al_2_O_3_ /wt%	Fe_2_O_3_ /wt%	K_2_O /wt%	CaO /wt%	MgO /wt%	Na_2_O /wt%
Initial	56.95	29.16	4.35	2.32	2.04	1.89	1.27
After	95.53	2.62	0.13	0.40	0.031	0.039	0.16

**Table 3:** Proximate analysis and elemental analysis of the carbonized waste tires.

Sample	Proximate analysis (wt%)																			
Carbonized waste tires	M_ad_					A_ad_					V_ad_					FC_ad_				
	0.705					18.69					1.765					78.84				
	Elemental analysis (wt%)																			
	C				H				O				N				S			
	78.44				0.437				4.351				0.39				3.51			

**Table 4:** Proximate analysis and elemental analysis of the pre-treated kerosene co-refining residue.

Sample	Proximate analysis (wt%)																			
Pre-treated kerosene co-refining residue	M_ad_					A_ad_					V_ad_					FC_ad_				
	0.36					16.41					47.36					35.87				
	Elemental analysis (wt%)																			
	C				H				O				N				S			
	76.65				4.973				4.319				0.7				1.662			

**Table 5:** Microstructural parameters of the carbonized waste tires and pre-treated kerosene co-refining residue.

Sample	S_BET_ (m^2^/g)	V_total_ (cm^3^/g)	Average Pore Size/(nm)
Carbonized waste tires	62.07	0.34	27.75
Pre-treated kerosene co-refining residue	11.52	0.043	12.59

## Data Availability

The original contributions presented in this study are included in the article. Further inquiries can be directed to the corresponding author.
